# Low Risk of Occult Hepatitis B Infection among Vietnamese Blood Donors

**DOI:** 10.3390/pathogens11121524

**Published:** 2022-12-13

**Authors:** Tran Thanh Tung, Jürgen Schmid, Vu Xuan Nghia, Le Chi Cao, Le Thi Kieu Linh, Ikrormi Rungsung, Bui Tien Sy, Truong Nhat My, Nguyen Trong The, Nghiem Xuan Hoan, Christian G. Meyer, Heiner Wedemeyer, Peter G. Kremsner, Nguyen Linh Toan, Le Huu Song, C.-Thomas Bock, Thirumalaisamy P. Velavan

**Affiliations:** 1 Institute of Tropical Medicine, University of Tübingen, 72074 Tübingen, Germany; 2 Vietnamese-German Center for Medical Research (VG-CARE), Hanoi 10000, Vietnam; 3 108 Military Central Hospital, Hanoi 10000, Vietnam; 4 Department of Parasitology, Hue University of Medicine and Pharmacy (HUMP), Hue 52000, Vietnam; 5 Department of Gastroenterology, Hepatology and Endocrinology, Hannover Medical School, 30623 Hannover, Germany; 6 German Center for Infection Research, Partner Hannover-Braunschweig, 38104 Braunschweig, Germany; 7 Centre de Recherches Medicales de Lambarene, Lambaréné B.P. 242, Gabon; 8 Department of Pathophysiology, Vietnam Military Medical University, Hanoi 10000, Vietnam; 9 Division of Viral Gastroenteritis and Hepatitis Pathogens and Enteroviruses, Department of Infectious Diseases, Robert Koch Institute, 13353 Berlin, Germany

**Keywords:** occult hepatitis B, hepatitis B virus, Vietnam, blood donors, hepatitis B surface antigen

## Abstract

Occult hepatitis B infection (OBI) is characterized by the presence of low levels of hepatitis B virus (HBV) DNA and undetectable HBsAg in the blood. The prevalence of OBI in blood donors in Asia ranges from 0.013% (China) to 10.9% (Laos), with no data available from Vietnam so far. We aimed to investigate the prevalence of OBI among Vietnamese blood donors. A total of 623 (114 women and 509 men) HBsAg-negative blood donors were screened for anti-HBc and anti-HBs by ELISA assays. In addition, DNA from sera was isolated and nested PCR was performed for the HBV surface gene (S); a fragment of the S gene was then sequenced in positive samples. The results revealed that 39% (*n =* 242) of blood donors were positive for anti-HBc, and 70% (*n =* 434) were positive for anti-HBs, with 36% (*n =* 223) being positive for both anti-HBc and anti-HBs. In addition, 3% of blood donors (*n =* 19) were positive for anti-HBc only, and 34% (*n =* 211) had only anti-HBs as serological marker. A total of 27% (*n =* 170) were seronegative for any marker. Two of the blood donors (0.3%) were OBI-positive and sequencing revealed that HBV sequences belonged to HBV genotype B, which is the predominant genotype in Vietnam.

## 1. Introduction

Hepatitis B occurs worldwide, and according to the World Health Organization (WHO), 296 million people were living with the virus in 2019, resulting in more than 800,000 HBV-related fatalities [1]. Of note, WHO South-East Asia and the WHO Western Pacific Region are among the areas with highest infection rates and account for approximately half of global chronic infections [1,2].

Following infection, HBV-associated hepatitis has an incubation period of 28 to 180 days. In most cases, the virus causes a self-limited acute infection [3,4]. However, about 5% of infections do not resolve and develop into a chronic state of disease [5]. Long-term HBV chronic carriers are exposed to an increased risk of cirrhosis, liver failure, and hepatocellular carcinoma [6]. It has been estimated that 15-40% of chronically infected HBV patients will develop serious sequelae during their lifetime [7].

In recent decades, extensive HBV vaccine implementation and improvements in hygiene, healthcare, and diagnostics have greatly reduced the risk of HBV infection. Nevertheless, HBV still remains among the most common posttransfusion infections today, as donors with occult HBV infection may be misdiagnosed, representing risks for the blood recipients [8,9,10]. Occult HBV infection (OBI) is defined as the presence of replication-competent HBV DNA (i.e., episomal HBV covalently closed circular DNA (cccDNA)) in the liver and/or HBV DNA in the blood of people who test negative for hepatitis B surface antigen (HBsAg) by currently available assays [11]. This phenomenon has been described for decades and its clinical implications have been recognized worldwide. However, the molecular mechanisms underlying OBI are not well characterized. In the post-window period, while HBV-DNA can be detected in low concentration, HBsAg maybe undetectable due to the resolution of the acute or chronic infection [12]. Alternatively, low S-gene expression or the presence of immune escape S-gene mutations in the “a” determinant and the Major Hydrophilic Region (MHR) have been postulated to cause false-negative HBsAg results [13]. Anyhow, it has been shown that OBI is associated with advanced chronic liver disease, and the virus remains transmissible in OBI cases [14,15].

In line with WHO’s Sustainable Development Goal 3, which calls for sustained action to eliminate viral hepatitis infections by 2030 [16], some developed countries have now required to assess the combination of HBsAg, anti-HBc, and HBV DNA detection in order to allow the diagnosis of the window period, as well as of occult infections [17]. However, in resource-limited settings, HBV screening in blood donors still relies on HBsAg tests alone, which can mask the true occurrence of OBI and lead to unintentional transmission of HBV via blood transfusion. Therefore, there is an urgent need to determine the prevalence of OBI in blood donors to fully understand the risk of transfusion-related HBV infections and to implement screening strategies accordingly, especially in HBV endemic regions. 

Vietnam, despite mandatory HBV vaccination policy from 2003, is still among the countries with a high burden of viral hepatitis. The rate of HBV infections in the Vietnamese population has been estimated to range from 8 to 13.3% [18], in particular in rural areas and older age groups [19,20]. Furthermore, previous studies have shown that the HBV genotypes B and C are dominant and occur in 75 % and 25 % of cases, respectively [21,22,23]. Nevertheless, reliable data on the occurrence of OBI among blood donors in Vietnam are still lacking.

In the present study, we use serology and molecular tests to investigate the prevalence and genotypes of OBI in HBsAg-screened blood samples from blood donors in Hanoi, Vietnam. The results from this study can help assess the risk factors for OBI transmission in the Vietnamese population.

## 2. Materials and Methods

### 2.1. Study Cohort

**Figure 1:**
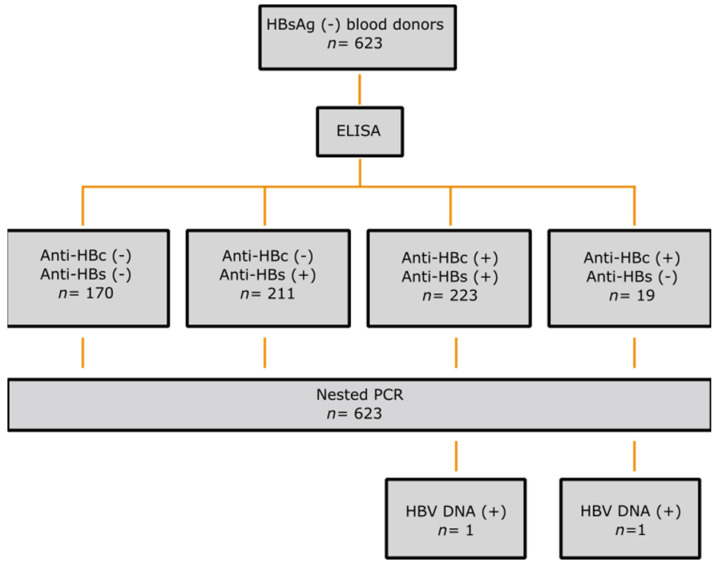
Study design and summary of results. A total of 623 HBsAg (-) blood donors were screened for anti-HBc and anti-HBs by ELISA before nested PCR targeting a highly conserved S/P region of the HBV genome. Two samples were found to be HBV DNA (+).

This cross-sectional study was carried out at the Department of Blood Transfusion, 108 Military Central Hospital, Hanoi, Vietnam. For this study, a total of 623 HBsAg-negative serum samples were collected from blood donors on 2 October 2021 and 12 October 2021. The study cohort was collected on two independent blood donation days, as mentioned above. All healthy adult volunteers from northern Vietnam were representative of this cohort and were predominantly from the Kinh ethnic group. No chronic diseases were recorded in the medical history. However, eligibility for blood donation was determined by the doctors of the transfusion department based on their routine guidelines. Demographic data and written informed consent were obtained from all donors. In accordance with standard hospital practice, the samples were serologically tested for HIV, HCV, and HBV (HBsAg) and were confirmed negative (VITROS Immunodiagnostic Products HBsAg, Anti-HIV 1+2, Anti-HCV (Ortho-Clinical Diagnostic, Felindre Meadows, UK). All blood donor samples were additionally tested for anti-HBs and anti-HBc antibodies and HBV DNA (Figure 1).

### 2.2. Serological Assays

The blood donor serum samples were screened for anti-HBs and anti-HBc using Monolisa^TM^ Anti-HBs PLUS and Monolisa^TM^ Anti-HBc PLUS (BIO-RAD, Hercules, CA, USA) ELISA kits according to the manufacturer’s instructions. Absorbance was measured using a CLARIOstar microplate reader (BMG Labtech, Ortenberg, Germany) and anti-HBs positivity was defined as a titer value >10 mIU/mL.

### 2.3. Nucleic Acid Isolation

Nucleic acid isolation from serum samples was done using QIAmp DNA Mini Kit (Qiagen GmbH, Hilden, Germany) according to the manufacturer’s protocol. For the isolation, 200 µl of serum was used and the nucleic acids were eluted in 80 µL of elution buffer. The quality and quantity of the DNA were measured using NanoDrop™ (Thermo Fisher Scientific, Waltham, MA, USA) and stored at −80 °C until use.

### 2.4. HBV Screening and Sequencing

HBV DNA in serum samples was investigated using a nested PCR by targeting a highly conserved S/P region (332 bp) of the HBV genome as previously described [8,22]. Amplification reactions were carried out in 25 µL (1× PCR buffer, 0.2 mM dNTPs, 0.4 µM specific primer, and 1U HotStarTaq DNA Polymerase (Qiagen GmbH, Hilden, Germany). Primers HBV-022, HBV-065, and HBV-066 were used for outer PCR, while primers HBV-024, HBV-041, and HBV-064 were used for inner PCR (Table S1). The thermal cycling program for the outer PCR: initial denaturation at 95 °C for 15 mins; followed by 35 cycles of denaturation (94 °C, 30s), annealing (55 °C, 30s), and extension (72 °C, 30s); and lastly a final extension of 5 mins at 72 °C. For the inner PCR, the thermal parameters remained the same, except for the annealing step being at 50 °C (30s) and the number of cycles being reduced to 30. A positive control (HBV plasmid DNA) and a negative control of the master mix were integrated to each run to validate the PCR products that produce a 340 bp fragment. The detection limit of HBV DNA by nested PCR was approximately 2.5 copies per reaction (between 30 and 40 copies/mL) [8].

Amplicons were checked by agarose gel electroporation, and the positive samples were purified and cleaned using ExoSAP-IT PCR (Thermo Fisher Scientific, USA) and subsequently sequenced using the BigDye^TM^ Terminator v.3.1 Cycle Sequencing Kit (Thermo Fisher Scientific, USA) on an Applied Biosystems 3130xl Genetic Analyzer (Thermo Fisher Scientific, USA).

### 2.5. Statistical and Phylogenetic Analysis

The illustration for age distribution was generated using R version 4.0 (http://www.r-project.org accessed on 4 October 2022). The sequences were trimmed in BioEdit Ver.7.2.5 (https://bioedit.software.informer.com/7.2/ accessed on 4 October 2022) and the resulting consensus sequences were aligned with known HBV B sequences from the Hepatitis B Virus database (HBVdb) online (https://hbvdb.lyon.inserm.fr/HBVdb/ accessed on 25 October 2022). Phylogenetic analysis was performed using Mega 11 (https://www.megasoftware.net/download_form accessed on 25 October 2022). The phylogenetic tree was constructed using the maximum likelihood method by using 52 HBV genomes randomly selected from the NCBI database representing all the genotypes. The tree was constructed using the model with the lowest BIC score (Table S2) and a bootstrap value of 1000. In our case, the GTR+G+I model was the best possible approximation. The two sequences were submitted to GenBank and were assigned accession numbers: OP038923 and OP038924.

## 3. Results

### 3.1. Baseline Characteristics

A total of 623 sera were collected from potential blood donors at the Military Central Hospital 108 in Hanoi. All donors were from the urban areas of the city and had a median age of 33 years (IQR = 29; 38.5). Male participants accounted for 82% of the cohort (*n* = 509).

### 3.2. HBV Serology and Nucleic Acid Detection

The sera were transported to the Institute of Tropical Medicine in Tübingen, Germany for serologic assays and HBV-DNA detection. The results (Table 1, Figure 1) showed that 27.3% (*n* = 170) were negative for both anti-HBs and anti-HBc antibodies, indicating a susceptible population, while 70% (*n* = 434) were positive for anti-HBs antibodies alone, indicating immunity due to vaccination [24]. On the other hand, 3.1% (n = 19) were positive for anti-HBc alone and 35.8% (*n* = 223) were positive for both anti-HBc and anti-HBs antibodies, suggesting prior infection [24].

**Figure 2:**
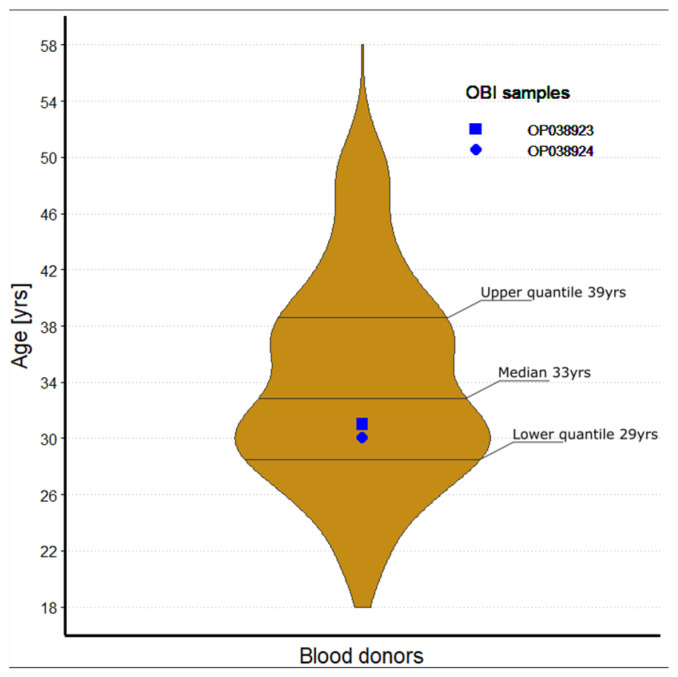
Age distribution of 623 blood donors; Including the upper quantile (39 y), the lower quantile (29 y) and the median (33 y). Both samples positive for OBI were marked in the graph OP038923 (30 y) and OP038924 (31 y).

We found by nested PCR that HBV DNA was present in two samples (0.3%; in OP038923 and OP038924). The OBI positive blood donors were 31 and 30 years old (Figure 2). As expected, both OBI carriers were seropositive and had anti-HBc antibodies; in addition, the OP038924 individual was positive for anti-HBs. It is evident that OBI is more likely to be found in people with anti-HBc positive but anti-HB-negative serology profiles [25,26], however, we found only 1 OBI positive case out of 19 samples in this group (Figure 1).

### 3.3. OBI Genotyping

**Figure 3:**
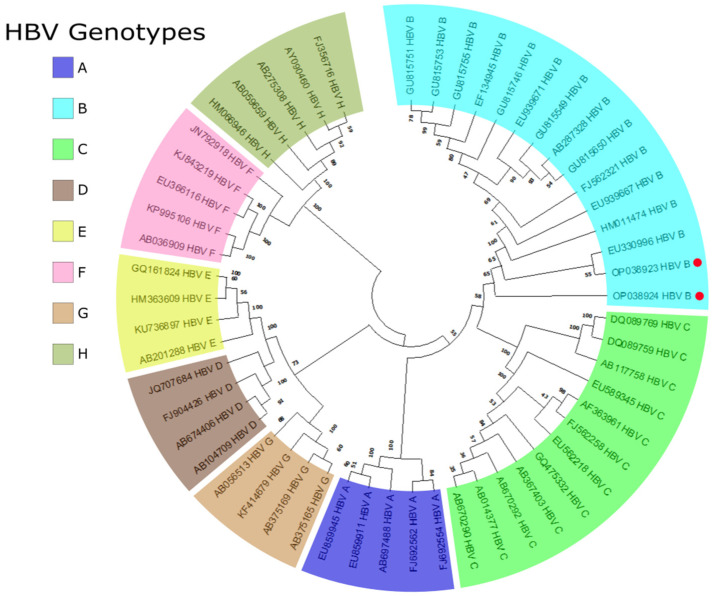
Reconstructed phylogenetic tree of the S gene from HBV using the GTR+G+I model, with 52 randomly selected whole genome sequences of A-H genotypes from the HBVdB. The OBI sequences from this study are marked with a red dot. Both sequences are in the genotype B clade.

After nested PCR, amplicons were purified and sequenced. BLAST searches against the NCBI database revealed that the two OBI cases belonged to HBV genotype B, which is predominant in Vietnam [21,22,23]. A phylogenetic tree was then constructed using the sequences of the two OBI positive samples and 52 representative S/preS HBV sequences of A-H genotypes from the HBV database (HBVdb), which showed the two OBI positive samples clustering with genotype group B, consistent with the BLAST search results (Figure 3).

**Figure 4:**
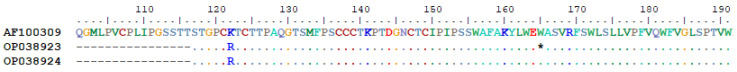
Surface protein alignment of the two OBI positive samples with the reference sequence AF100309 from the HBVdb. The alignment shows aa 100 to 191 of the S gene. The MHR spans aa 99–169 and the "a" determinant span aa 124–147. The amplicon starts from aa 117. Sequences shown as a dot have the same aa as the reference sequence; only variations are shown. An asterisk indicates that the sequence was terminated by a transcribed stop codon.

**Figure 5:**
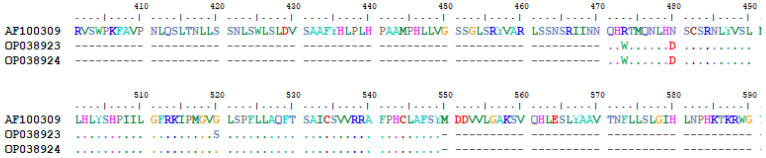
Polymerase protein alignment of the two OBI positive samples with the reference sequence AF100309 from the HBVdb. The alignment shows aa 400 to 591 of the P gene. Sequences shown as a dot have the same amino acids as the reference sequence; only variations are shown. An asterisk indicates termination due to a transcribed stop codon.

Multiple nucleotide mutations were identified, using sequence AF100309 from HBVdb as the reference. Nucleotide substitutions C510T, A521G, and A531G were observed in both Vietnamese OBI carriers, while G651A was only observed in OP038923. Due to the overlapping nature of the viral Open Reading Frames (ORFs), these single nucleotide polymorphisms lead to five amino acid changes in both the S (Reading Frame 1) and the P (Reading Frame 2) genes. For the S gene, mutation K122R in the MHR was observed for both samples, while W165X, resulting in a stop codon, was only present in OP038923 (Figure 4). For the P gene, three amino acid changes were observed, R473W and N480D in both samples and G520S only in OP038923 (Figure 5).

## 4. Discussion

To eliminate the spread of HBV through vertical or horizontal transmission, the diagnosis of OBI among blood donors is of importance. In general, OBI prevalence varies considerably by regions, genotypes, molecular methods used, detection limits of HBsAg tests, and risk groups [8,11,27]. In Laos, a prevalence of 10.9% was reported among HBsAg-negative blood donors [28], while a study from China found a low OBI prevalence of 0.013% only [29]. In eastern India, OBI cases were found in 2.96% of blood donors [30], and in Nigeria, a 17% OBI prevalence was reported in a population of 429 blood donors [8]. In the present study, in a study population of 623 HBsAg-negative blood donors from Hanoi, we found an OBI prevalence of 0.3%, which is low compared to the number of cases reported in a literature review for Asia (0.013–5.5%) [27,31]. This is noteworthy considering the fact that Vietnam is highly endemic for HBV [19,22], which is confirmed by the high seroprevalence of HBV markers found in this study. In fact, 73% of our samples tested positive for at least one HBV antibody (anti-HBs and anti-HBc), with 39% being positive for anti-HBc. In addition, a recent meta-analysis indicated that OBI prevalence in blood donors in highly endemic regions ranged between 0.44 and 1.72% [12]. However, it is important to point out that these data are largely derived from studies on Chinese populations, which may not be predictive of the OBI prevalence in Vietnam. Low levels of OBI could also be due to the sensitivity of the HBsAg test used, or good health care that reduces the risk of OBI transmission [32,33]. Furthermore, regionality and socioeconomic status have been shown to influence the discrepancy in HBV infections and OBI rates between populations [34]. The cohort in the present study was from the capital of Vietnam, which has more access to vaccination, improved health care access, and higher socioeconomic status. Therefore, the OBI rate in Vietnam is likely higher in the general population and even higher in rural regions. It is also important to note that the analytical sensitivity of the molecular tests for HBV DNA may have had an impact on the results of this study and may be another source of discrepancy in the prevalence of OBI in different studies.

In Asia, HBV genotypes B and C are common [21] and approximately 75% of all HBV infections in Vietnam are caused by genotype B [21,35], which is the genotype identified in both our OBI-positive individuals. Of note, HBV group B has been particularly associated with immune escape variants and early viral load reduction due to early HBeAg clearance [36]. It is also noteworthy that many HBV immune escape and OBI-associated virus variants harbor mutations in the S gene, especially in the MHR [37,38,39]. Mutations in this region can alter the epitope binding site and reduce the efficacy of neutralizing antibodies [40], resulting in anti-HB-negative and anti-HBc-positive serology [41]. In addition, it has been shown that anti-HBc-positive and HBsAg-negative carriers may still have replicative HBV DNA due to highly stable covalently closed circular DNA or genome-integrated viral DNA [41,42]. The mutations in sample OP038923 (positive for anti-HBc alone) reflect the above observations. On the other hand, although none of the two OBI-positive samples had an amino acid substitution in the “a” determinant, OP038923 harbored a mutation in the MHR (W165X) that could lead to the early termination of HBsAg. As a result, the shorter S protein might have led to a structural change that reduced the immunogenicity of the epitope and possibly explains the subsequent loss of anti-HBsAg in ELISA assays or in the host. Alternatively, a dysfunction could have occurred, causing the S-proteins to not fully anchor in the envelope, reducing the viral load. On the other hand, the presence of HBV-DNA with anti-HBs and anti-HBc-double-positive profile could be explained by a resolved infection without complete elimination of the virus in the hepatocytes [43,44].

The substitution K122R in the S protein, which was present in both carriers, has previously been associated with OBI. An *in vitro* study investigated the effect of three different mutations, including K122R, on HBsAg secretion [45]. K122R alone had no effect on HBsAg secretion, but in combination with the other mutations, secretion was reduced. Furthermore, K122R changed the serotype from "d" to "y", possibly resulting in an anti-HBs escape variant [45,46]. Other studies mentioned that the amino acid substitution K122R is quite common in genotype B in Chinese and Vietnamese populations [47,48]. For the polymerase protein, both carriers harbored the amino acid substitution N480D in the non-epitope site of the protein. Wang et. al. reported this mutation in two patients with chronic HBV genotype B infection [49], which potentially affect the polymerase activity [41,50]. Of note, R473W, G520S and W165X have never been reported in polymerase or S protein, respectively. Contrary to the general assumption that OBI is related to mutations in the S gene, particularly in the MHR [37], 40% of the mutations found in the present study are in the MHR and 60% in the polymerase gene. However, it is also important to note that this study is limited to a 239 bp sequence of the genome, and sequencing of the whole viral genome could reveal mutations in other regions that may have an impact. Another limitation of the present study is that the viral loads of OBI-positive individuals cannot be quantified.

## 5. Conclusions

In conclusion, this study focused on molecular and serological screening of sera from Vietnamese blood donors in order to identify OBIs. Despite the high anti-HBc positivity rate, we found a low prevalence of OBI (0.3%) in 623 blood donors. Both OBI-HBV DNA showed typical mutations in the surface antigen gene. These results demonstrate the importance of molecular testing in preventing the spread and reactivation of HBV in immunocompromised patients and high-risk groups; and supports the introduction of molecular tests in accordance with WHO’s goal of eradicating HBV by 2030. Further studies in different provinces of Vietnam will help to determine the relative risk of transfusion-transmitted HBV infection in the Vietnamese population in the coming years.

## Figures and Tables

**Table 1:** Overall prevalence of anti-HBs and anti-HBc markers and combination.

Serology	Number (%)
Anti-HBc-positive	242 (39)
Anti-HB-positive	434 (70)
Anti-HB-negative and anti-HBc-positive	19 (3)
Anti-HB-positive and anti-HBc-negative	211 (34)
Anti-HB-positive and anti-HBc-positive	223 (36)
Anti-HB-negative and anti-HBc-negative	170 (27)

## Data Availability

The sequences of the S gene fragment from patients OP038923 and OP038924 are available on GenBank under the same accession numbers.

## Supplementary Materials

The following supporting information can be downloaded at: https://www.mdpi.com/article/10.3390/pathogens11121524/s1, Table S1: Primer sequences for HBV genotyping; Table S2: MEGA11 calculated parameter model for phylogenetic tree construction.
