# Supplementary material for: Low Risk of Occult Hepatitis B Infection among Vietnamese Blood Donors
Source: Pathogens. 2022 Dec 13;11(12):1524. doi: 10.3390/pathogens11121524 (PMC9786887; doi:10.3390/pathogens11121524)
Supplement: Supplementary file 1 [file pathogens-11-01524-s001.zip › pathogens-2080158-supplementary.pdf]

Table S1. Primer sequences for HBV genotyping.

Primer	Sequence (5′ – 3′)
HBV-022	TGCTGCTATGCCTCATCTTC
HBV-065	CACAGATAACAAAAAATTGG
HBV-066	CAAAGACAAAAGAAAATTGG
HBV-024	CAAGGTATGTTGCCCGTTTGTCCT
HBV-041	GGACTCAMGATGYTGCACAG
HBV-064	GGACTCACGATGCTGTACAG

Primer 22,65,66 are used for outer PCR and 24,41,64 for inner PCR

Table S2. MEGA11 calculated parameter model for phylogenetic tree construction.

Model	#Param	BIC
GTR+G+I	115	43875,22159
GTR+G	114	43901,52895
HKY+G+I	111	44001,49089
TN93+G+I	112	44004,5065
K2+G+I	108	44011,90295
T92+G+I	109	44013,23805
HKY+G	110	44014,5537
TN93+G	111	44015,32172
K2+G	107	44025,263
T92+G	108	44026,81607
GTR+I	114	44208,39446
HKY+I	110	44296,81056
TN93+I	111	44301,57063
K2+I	107	44306,5673
T92+I	108	44308,25793
JC+G+I	107	44919,28712
JC+G	106	44928,83522
JC+I	106	45192,69854
GTR	113	46778,54906
TN93	110	46915,54949
HKY	109	46916,86083
K2	106	46920,15131
T92	107	46926,47696
JC	105	47766,69872
